# Assessing proton plans with three different beam delivery systems versus photon plans for head and neck tumors

**DOI:** 10.1002/acm2.70013

**Published:** 2025-02-17

**Authors:** Tara Gray, Chieh‐Wen Liu, Saeed Ahmed, Anna Maria Kolano, Jeremy Donaghue, Shlomo Koyfman, Neil Woody, Shauna R. Campbell, Jonathan B. Farr, Ping Xia

**Affiliations:** ^1^ Department of Radiation Oncology Cleveland Clinic Foundation Cleveland Ohio USA; ^2^ Department of Radiation Oncology University of Kansas Medical Center Kansas City Kansas USA; ^3^ Applications of Detectors and Accelerators to Medicine (ADAM) Advanced Oncotherapy (AVO) plc London UK

**Keywords:** head and neck cancer, IMPT, proton therapy, radiation therapy

## Abstract

**Purpose:**

To compare plan quality among photon volumetric modulated arc therapy (VMAT) and intensity‐modulated proton therapy (IMPT) with robustness using three different proton beam delivery systems with various spot size (σ) ranges: cyclotron‐generated proton beams (CPBs) (σ: 2.7–7.0 mm), linear accelerator proton beams (LPBs) (σ: 2.9–5.5 mm), and linear accelerator proton mini beams (LPMBs) (σ: 0.8–3.9 mm) for the treatment of head and neck (HN) cancer with bilateral neck irradiation.

**Methods:**

Ten patients treated for oropharynx cancer with bilateral neck irradiation were planned using CPBs, LPBs, LPMBs, and VMAT. The homogeneity index (HI), mean body dose, and defined volumetric doses for selected critical organs‐at‐risk (OARs) were compared. Set‐up uncertainties of ±3 mm and ± 3.5% range uncertainties were included in robust evaluation using V_95%Rx _> 95% (Volume that covers 95% of the target volume at 95% of the prescription (Rx) dose) to high dose and low dose CTV volumes (CTV_70 Gy and CTV_56 Gy). VMAT and proton plans were compared in terms of OAR doses and mean body dose only. Homogeneity Indices were compared among IMPT plans in addition to OAR doses. The Wilcoxon signed‐rank test was used to evaluate statistical differences between evaluation metrics for VMAT plans and all proton plan types.

**Results:**

OAR dose metrics were improved by 2% to 30% from CPB plans to LPB or LPMB plans. Compared to photon VMAT plans, all OAR doses except for mandible dose metrics were improved by 2% to 53% for all proton plans. The mean body dose was also improved by 7.5% from CPB to LPB and by 10.8% from CPB to LPMB. In addition, the mean body dose was also improved by 44% from VMAT to CPB, by 48% from VMAT to LPB, and by 50% from VMAT to LPMB plans. Compared to CPB plans, HI was significantly better (*p* < 0.05) for the LPB and LPMB plans. HI also improved considerably from VMAT to CPB, LPB, and LPMB. For both CTV_70 Gy and CTV_56 Gy, average robust evaluation across all worst‐case scenarios was slightly better for CPB plans, with an average of V_95%Rx_ of the CTV_70 Gy of 97.6% ± 1.22%, followed by 97.2% ± 1.31% and 97.2% ± 1.35% for LPB and LPMB plans, respectively. Robustness for CTV_56 Gy showed comparable robustness across all proton plan types, with an average V_95%Rx_ of 97.4% ± 0.87% for CPB, 97.4% ± 1.21%, and 97.5% ± 1.08% for CPB, LPB, and LPMB plans, respectively.

**Conclusion:**

With decreased spot size, the LPB and LPMB are excellent alternatives to VMAT and CPB therapy and can significantly reduce the dose to normal tissue.

## 1 INTRODUCTION

Head and Neck (HN) cancers are typically treated using volumetric modulated arc radiation therapy (VMAT). However, many radiation therapy‐related toxicity concerns, such as acute grade 2 and 3 toxicity, still exist, warranting new treatment strategies to mitigate this risk.^1^ Using proton therapy, especially intensity‐modulated proton therapy (IMPT), to treat HN cancer can increase the radiation dose to the tumor while simultaneously decreasing the radiation dose to surrounding healthy tissue. Many previous studies show that proton therapy can deliver highly conformal radiation dose to the target while still sparing normal tissue.^2^, ^3^ Although evidence is still accruing on long‐term survival outcomes comparing photons versus protons,^4^ data suggests that proton therapy can better spare surrounding healthy tissue and thus has lower rates of adverse events.^1^, ^5^ With the introduction of IMPT, clinical results from proton therapy have further improved.^5^, ^6^, ^7^, ^8^, ^9^, ^10^ IMPT is a sophisticated proton delivery technology that allows for greater degrees of freedom to produce optimized dose distributions, which is essential when treating large volumes, such as primary HN sites with simultaneous ipsilateral or bilateral neck coverage.^11^ Many studies on the use of IMPT for HN cancer also show its superior normal tissue sparing and biological advantages, such as improved tumor control probability (TCP) and normal tissue complication probability (NTCP), when compared with standard photon treatments.^8^, ^12^, ^13^ IMPT has several advantages over passively scattered proton therapy (PSPT) and intensity‐modulated photon therapy (IMRT) for HN cancer.^8^, ^11^, ^14^, ^15^, ^16^, ^17^, ^18^, ^19^, ^20^ Compared to PSPT, IMPT relies on electromagnetic control of the pencil beam to achieve conformal dose distribution while reducing the use of patient‐specific devices such as compensators, which are labor intensive to produce, prolong treatment time, and increase scatter. Compared to IMRT, IMPT reduces the integral dose bath while achieving similar dose conformity.^11^ The disadvantage of IMPT is its high sensitivity to radiologic density changes due to anatomical changes, organ motion, or patient motion.^11^

The benefits of IMPT can also be significantly improved with new proton beam delivery systems. Some newer technologies for proton therapy include the development of linear accelerator‐based proton therapy.^21^, ^22^ Currently, most commercially available proton beam systems employ therapeutic beams with energies ranging from 70 to 230 MeV, capable of penetrating a water‐equivalent thickness of 4–32 cm. High‐quality IMPT plans can be achieved using a small number (3 to 4) of fields.^11^ Over the years, many innovations to proton therapy have been made, such as new treatment technologies, including linear accelerator proton machines, enabling smaller spot sizes and thus, sharper dose fall‐off^23^ and better sparing of normal tissues.^24^ Compared with standard cyclotron‐generated proton beams (CPBs), where protons are accelerated in a spiral pattern, a linear accelerator‐based proton beam accelerates protons in a straight line, produces a better‐controlled spot size with each energy, and utilizes electronic energy control, enabling fast energy switching pulse by pulse.^22^, ^25^ A proton linear accelerator also has small emittance, resulting in smaller spot sizes and low beam losses, requiring less shielding than CPBs. In contrast to cyclotrons, a proton linac uses multiple radiofrequency cavities to accelerate particles in a straight path with a beam produced in pulses. With the added feature of producing proton mini beams with sub‐millimeter spot sizes, the proton linear accelerator becomes an attractive alternative to photon therapy and CPB therapy. In addition, unlike the spot enlargement observed with decreasing energy for cyclotrons, the proton linac spot size is invariant (constant) between energies of 150–230 MeV, rendering proton linac treatment plans more conformal than cyclotron‐based plans.^22^, ^26^ In the present study, spot sizes (defined in air) varied from 7 to 2.7 mm for CPB, decreasing as the energy increased from 70 to 230 MV. For the linear accelerator proton beam (LPB) beamline, the spot sizes ranged from 5.5 to 2.9 mm, decreased from 70 to 150 Mev and remained constant after 150 MeV. For the LPB beamline, the spot size (defined in air) varied from 3.9 to 0.9 mm, decreasing as the energy increased from 70 to 150 MeV and remained constant after 150 MeV.

In this study, we compare plan quality and robustness among photon VMAT and IMPT using three types of beam delivery systems: CPBs, LPBs, and linear accelerator proton mini beams (LPMBs), for the treatment of HN cancers. The use of the new beam delivery system machine models (LPB and LPMB) and comparing large and small spot sizes for planning HN cases is the major innovation in this particular study.

## 2 MATERIALS AND METHODS

### 2.1 Patient information and plan set‐up

The study is approved by a local institutional research board (IRB). A total of ten patients with T1‐T2 primary tumors of the oropharynx, five each of base of tongue and tonsil, were included in this study. Most patients (70%) had ipsilateral pathologic adenopathy, and 30% had bilateral disease. All patients were treated with definitive radiotherapy consisting of 70 Gy in 35 fractions to the primary tumor and involved lymph node(s) with simultaneous integration treatment of bilateral elective lymph nodes to 56 Gy. Each patient dataset consisted of a planning CT, corresponding targets, and critical structures.

For each patient, four plans were generated for this research, including:

1. A VMAT photon‐based plan.
2. A standard cyclotron‐based proton plan (CPB) using a common cyclotron beam model.
3. A proton plan generated using the LIGHT (Linear accelerator for Image‐Guided Hadron Therapy,^27^ Advanced Oncotherapy, London, UK and Applications of Detectors and Accelerators to Medicine, Meyrin, Switzerland) beam model (LPB).
4. A proton plan generated using the LIGHT mini‐beam model (LPMB).

### 2.2 VMAT photon plans

All VMAT photon plans were generated in Raystation (Version 11B, Raysearch Labs, Stockholm) using the collapsed cone convolution algorithm and a grid resolution of 3 mm. All patient plans consisted of the primary tumor and bilateral neck nodes. The clinical target volumes were expanded by 3 mm to generate a high‐dose primary tumor volume (PTV_70 Gy) for primary tumors and a low‐dose tumor volume (PTV_56 Gy) for elective nodal irradiation. The delineated tumor volumes and organs‐at‐risk (OARs) were based on institutional protocol.^28^ The plans were generated using the VMAT‐based simultaneous integrated boost planning technique. In each VMAT plan, three full arcs were used with 6 MV beams from the Varian Truebeam Linear accelerator equipped with standard 120‐leaf Millennium MLC. The collimator was slightly rotated by ± 10° for the first two arcs and by 90° for the third arc. The planning goals followed institutional criteria to deliver the prescription doses to ≥95% of both PTVs. The criteria for target coverage and normal tissue constraints are listed in Table 1.^28^

**TABLE 1:** OAR plan acceptance criteria for HN planning.

OAR	Plan acceptance criteria
PTV_70 Gy	V_Rx_ ≥ 95%
PTV_56 Gy	V_Rx_ ≥ 95%
Spinal cord	D_max_ < 45 Gy
parotids	D_mean_ < 26 Gy
Larynx	D_mean_ < 35 Gy
Mandible	D_1cc < _70.0 Gy
Trachea	D_mean_ < 45 Gy
Lips	D_mean_ < 20 Gy
Oral cavity	D_mean_ < 35 Gy
Submandibular glands	D_mean_ < 39 Gy
Oarpharynx	D_mean_ < 45 Gy

### 2.3 IMPT proton plans

**FIGURE 1:**
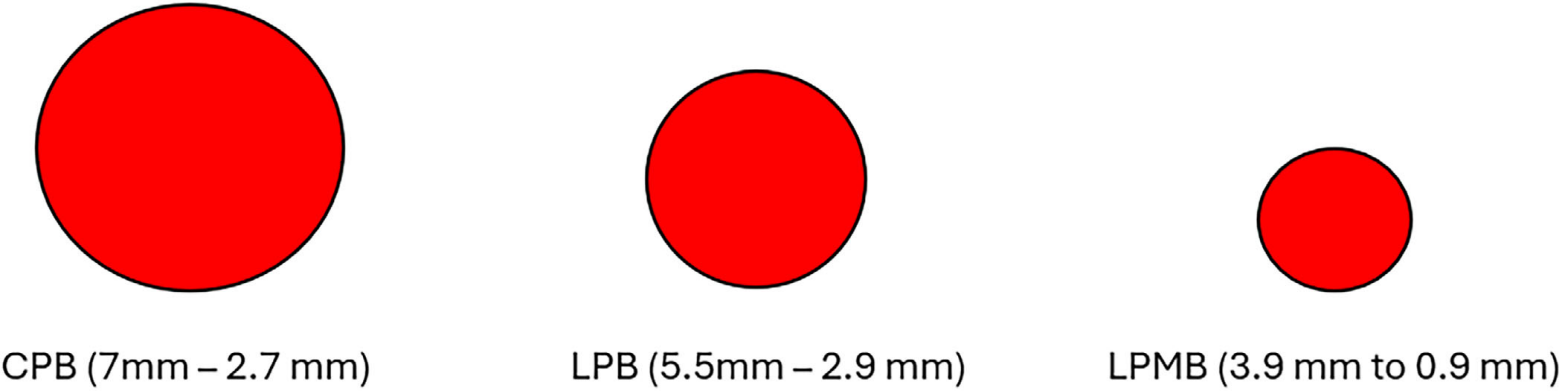
Relative σ for CPB, LPB and LPMB beam lines. CPB, cyclotron‐generated proton beams; LPB, linear accelerator proton beams; LPMBs, linear accelerator proton mini beams.

All proton plans were also generated in Raystation using the IMPT technique with multi‐field optimization (MFO). A typical proton pencil beam scanning machine with energies ranging from 70–230 MeV was used for CPB plans. A linear proton beam accelerator machine with energies from 30–230 MeV was used for LPB and LPMB plans. All proton plan types differed in spot sizes between 230 and 30 MeV, respectively, with σ of 2.7–7.0 mm for CPB plans, σ of 2.9–5.5 mm for LPB plans, and σ of 0.9–3.9 mm for LPMB plans. Each spot represents the lateral spatial distribution from a single static beamlet and is expressed in terms of beam sigma (σ) of the Gaussian distribution, defined in‐air at the isocenter for a particular energy. A diagram of the relative spot sizes is shown in Figure 1. Characteristics of the CPB versus the LIGHT system are described in Table 2. All proton doses are reported as Gy[RBE] using RBE = 1.1.

**TABLE 2:** CPB versus LIGHT system.

	CPB	Light
Acceleration direction	Spiral	Straight line
Pulsed beam?	No	Yes
Beam emittance	3.0–9.0	0.25
Energy modulation	Only with degrader‐absorbers	By electronic control
Proton losses, activation, and time structure	High in ESS	Low
Change of the energy (speed)	80 ms to 2.1 s	5 ms

**FIGURE 2:**
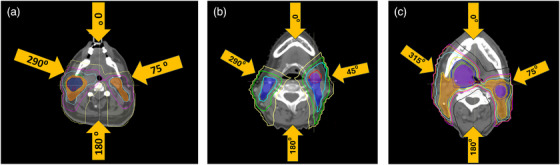
Example gantry angles used for each patient. (a) 290 and 75 degree angles and (b) 290 and 45 degree angles and (c) 315 and 75 degree angles. LPMBs, linear accelerator proton mini beams; VMAT, volumetric modulated arc therapy.

For IMPT plans, four beams were used per plan: 0°, 180°, and two anterior oblique beams using the IEC (International Electrotechnical Commission) convention. Anterior oblique beams ranged between 290° and 315° and 45° and 75° for the right and left sides of the patient, respectively, and were selected depending on tumor location for each plan. Figure 2 shows an example of the beam angles used for each patient in the study cohort. Range shifters of 4 cm were applied to each beam and used to pull the energy range to the desired treatment depth. The energy layer spacing, spot spacing, and lateral margin spacing were automatically determined by the treatment planning system (TPS), setting the “automatic with scale” to 0.5 for each of these parameters. Under the “automatic with scale” setting for spot spacing, the spot spacing is determined as 1.06 times the average spot size (1σ) at the Bragg peak depth in the patient, multiplied by the user‐defined scaling constant, defined as 0.5 in our particular case. This means that the spot spacing was defined as 0.5σ at the Bragg peak maximum in water. For the “automatic with scale” option for energy layer spacing, a variable distance that depends on the Bragg peak width is defined and multiplied by a scaling factor. The energy separation between two adjacent energy layers equals the energy loss over the width (80% dose level) of the most distal Bragg peak in the pair. This means that each Bragg peak will intersect the following Bragg peak at approximately 80% of the dose maximum for a scaling factor of 1. For the purposes of this study, energy layer spacing was scaled using a user‐defined factor of 0.5. A lateral margin is also needed for full target coverage without creating hot spots at the target border, but if a higher dose at the target edge can be tolerated, a target margin of 0 can be used to minimize lateral penumbra. If the “automatic with scale” option is selected for the lateral margins, the lateral target margin is determined as a function of the average spot size at the Bragg Peak maximum for the highest energy. In this study, a scaling constant of 0.5 was used as well, defining the lateral margin as 0.5σ at the Bragg Peak maximum at the highest energy.^29^ With robust optimization, additional target margins are automatically added to user‐defined uncertainty values.^30^ A MFO planning technique was used for each case. Avoidance structures were constructed and added to the OAR range margin in Raystation to avoid delivering the dose to the right shoulder and left shoulder. Another avoidance structure was created posteriorly to avoid giving excessive dose to the spine and other sensitive structures from the 180° field. These structures were constrained to keep the surrounding dose as low as reasonably achievable. Targets were split between right and left in “Plan Optimization,” and spots were assigned to the left side from the left side beam and the right side from the right side beam. Spots were assigned from the anterior beam only to the posterior part of the targets to avoid assigning dose to sensitive structures that beam might pass through. CTV‐based robust optimization with 3 mm set‐up uncertainty in all directions and 3.5% range uncertainty was applied to CTV_70 Gy and CTV_56 Gy for all plans. For the nominal scenario, all proton plans had V_100%Rx_ of each CTV_70 Gy, each CTV_56 Gy > 98%, and V_100%Rx_ of the GTV > 99%. In the voxel‐wise worst‐case scenario, the nominal proton plans were normalized to have at least V_95%Rx_ of all CTVs > 95%. Final dose calculation was performed for all plans using the Monte Carlo algorithm with a 0.3% uncertainty and a dose grid size of 3 mm.

### 2.4 Plan evaluation

To compare the plan quality among IMPT plans, the Homogeneity Index (HI), OAR doses, and mean body dose were assessed. The HI was defined as the maximum dose (D_max_) divided by the prescription dose (D_Rx_), D_max_/D_Rx_. The mean body dose was evaluated as the mean dose to the external contour of the patient. OAR doses were assessed based on the OAR plan acceptance criteria in Table 3. The paired Wilcoxon signed‐rank test was used to compare plan quality among VMAT plans and all proton plan types, with *p* < 0.05 considered statistically significant.

**TABLE 3:** OAR goals and doses for VMAT photon and proton plans.

OAR	Goal	VMAT (Gy)	Cyclotron protons (Gy)	Light beam protons (Gy)	Light mini beam protons (Gy)
**Spinal cord**	D_0.03 cc_ < 33 Gy	24.64	15.95	15.58	16.44
**R Parotid**	Mean dose < 26 Gy	20.86	19.23	18.71	18.34
**L Parotid**	Mean dose < 26 Gy	20.65	19.02	18.30	17.91
**Mandible**	D_1 cc_ < 70.0 Gy	64.20	64.67	62.90	62.26
**Oral cavity**	Mean dose < 35 Gy	31.30	18.91	16.93	16.26
**OARpharynx**	Mean dose < 45 Gy	43.90	32.45	29.81	28.60
**Larynx**	Mean dose < 30 Gy	22.03	14.62	11.92	10.28
**Integral dose**	Mean body dose (Gy)	8.02	4.47	4.14	3.99

## 3 RESULTS

**FIGURE 3:**
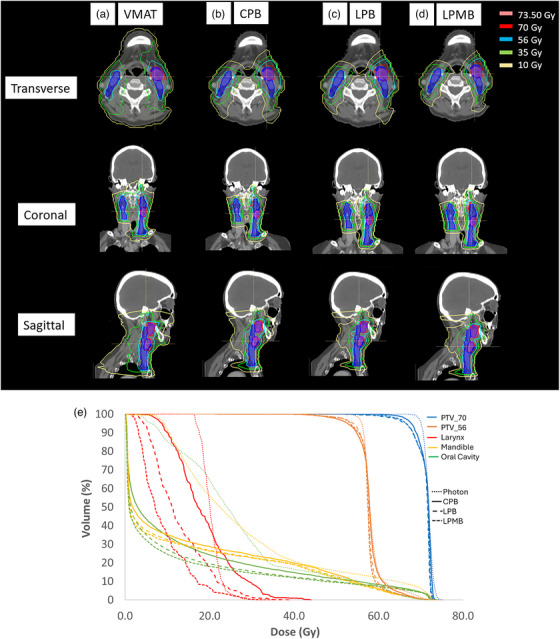
Axial, Sagittal, and Coronal views for HN plans: (a) VMAT dose distribution compared with (b) CPB, (c) LPB, and (d) LPMB proton plan types. For each view, the lower dose isodose lines conform better to the target volume going from left to right (VMAT to LPMB). The dose‐volume histogram for this specific case showing a comparison between photon, CPB, LPB, and LPMB for targets and specified OARs is shown in (e). This figure also shows how the dose distribution improves from VMAT to LPMB plans. The low dose is less prevalent, and dose fall‐off improves significantly for proton plans. CPB, cyclotron‐generated proton beams; LPB, linear accelerator proton beams; LPMBs, linear accelerator proton mini beams; VMAT, volumetric modulated arc therapy; HN, head and neck; OARs, organs‐at‐risk.

Figure 3 shows dose distributions among VMAT, CPB, LPB, and LPMB plan types for a selected case. Table 3 shows the dosimetric trend for specific endpoints for critical OARs and mean dose to the body. LPB and LPMB beamlines offer the lowest dose to these OARs. All quantities improve for proton plans when compared to VMAT plans, except for the average D_1cc_ to the mandible in CPB plans, which showed a 0.72% increase in the average D_1cc_ dose from VMAT. The mean dose to the larynx and oral cavity improved most from VMAT to IMPT plans. The mean dose to the larynx had a 46% improvement from the VMAT to LPB plans and a 53% improvement from VMAT to LPMB plans, while the mean dose to the oral cavity showed a 46% improvement from VMAT to LPB plans and a 48% improvement from VMAT to LPMB plans. The mean dose to the larynx had 19% improvement from CPB to LPB plans and a 28% improvement from CPB to LPMB plans. As shown in Table 3, all other dose metrics in OARs showed a 2% to 14% improvement from CPB to LPB or from LBP to LPMB plans. The mean body dose showed a 48% to a 50% improvement from VMAT to the LPB and LPMB plans and a 44% improvement in CPB plans from VMAT plans. All differences were significantly different, with a *p* < 0.05 for all OARs except for the mandible, which showed a *p* > 0.05 between VMAT and all proton modalities.

**FIGURE 4:**
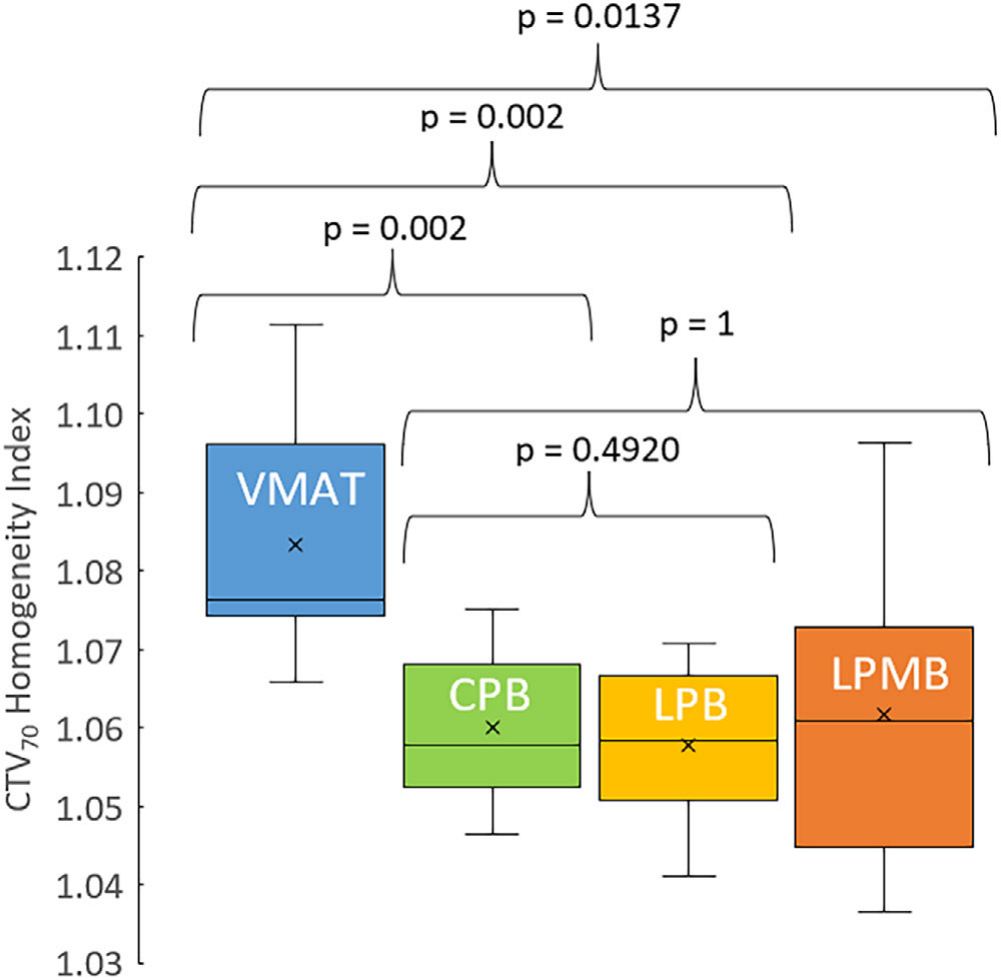
HI for VMAT versus CPB, LPB, and LPMB plans among 10 bilateral HN lesions. CPB, cyclotron‐generated proton beams; LPB, linear accelerator proton beams; LPMBs, linear accelerator proton mini beams; VMAT, volumetric modulated arc therapy; HI, homogeneity index; HN, head and neck; OARs, organs‐at‐risk.

Figure 4 shows the HI compared among proton plans for all 10 HN plans. The average HI and range of HI for VMAT plans were 1.08 ± 0.05 (1.07–1.11), 1.06 ± 0.01 (1.05–1.08) for CPB, 1.06 ± 0.01 (1.04–1.07) LPB plans, and 1.06 ± 1.02 (1.04–1.10) for LPMB plans.

**FIGURE 5:**
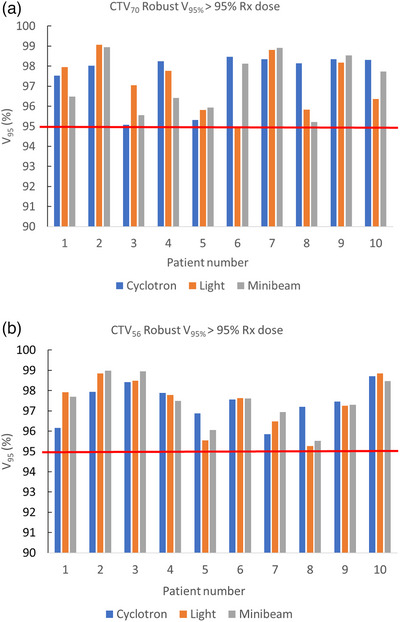
V_95%_ results for (a) CTV_70 Gy and (b) CTV_56 Gy robust planning for each patient and plan type. Each patient plan was planned and analyzed using 3 mm isotropic uncertainty and 3.5% density (statistical) uncertainty. V_95% _> D_95%Rx_ criteria. The red line defines passing criteria.

**FIGURE 6:**
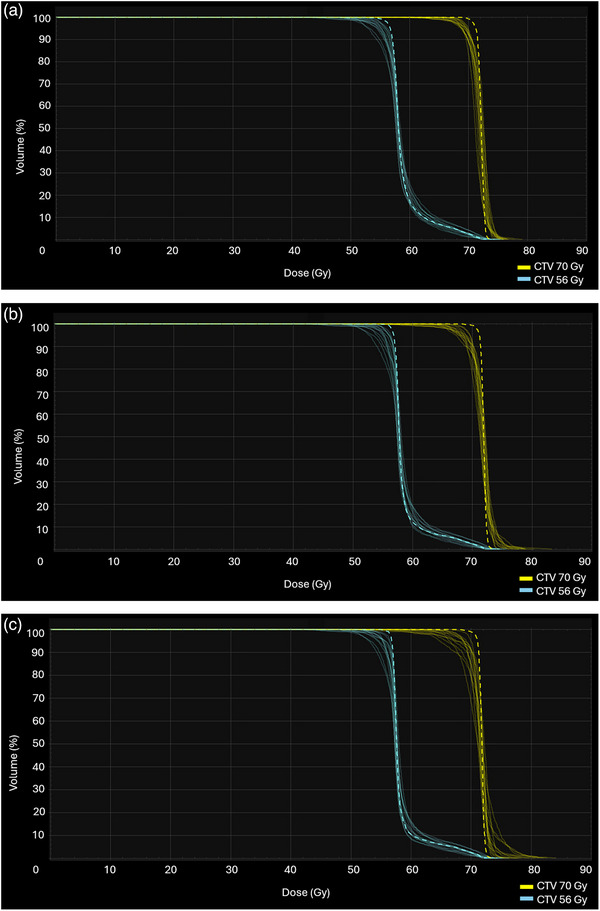
DVH bands of the CTV for each proton beam line for patient 10: (a) CPB, (b) LPB, and (c) LPMB. More robust plans, such as shown in (a) produce narrower DVH bands. The dashed line represents the CTV of the nominal plan. CPB, cyclotron‐generated proton beams; LPB, linear accelerator proton beams; LPMBs, linear accelerator proton mini beams.

**FIGURE 7:**
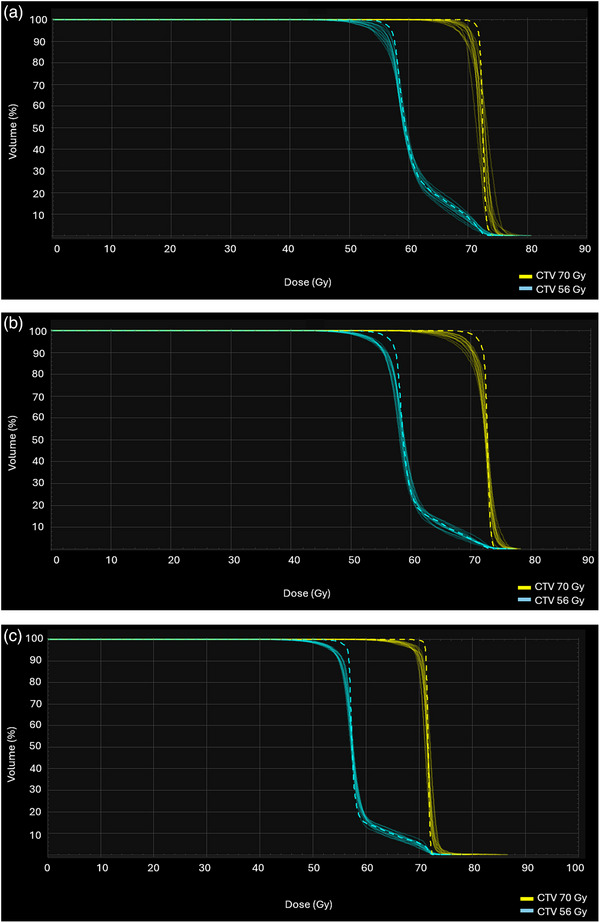
DVH bands of the CTV for each proton beam line for patient 2: (a) CPB, (b) LPB, and (c) LPMB. More robust plans, such as shown in (a) produce narrower DVH bands. The dashed line represents the CTV of the nominal plan. CPB, cyclotron‐generated proton beams; LPB, linear accelerator proton beams; LPMBs, linear accelerator proton mini beams.

Figure 5a,b show the V_95%_ for each proton plan type across the patient cohort. Average robust evaluation across all worst‐case scenarios was slightly better for CPB plans, with an average V_95%Rx_ of the CTV_70 Gy to be 97.6% ± 1.22% for CPB plans, followed by 97.2% ± 1.31% and 97.2% ± 1.35% for LPB and LPMB plans, respectively. Robustness for CTV_56 Gy showed a more consistent pattern across all proton plan types, with an average V_95%Rx_ of 97.4% ± 0.87%, 97.4% ± 1.21%, and 97.5% ± 1.08% for CPB, LPB and LPMB plans, respectively. All proton plans met robust metrics of V_95%Rx _> 95% prescription (Rx) dose. As shown in Figure 5a,b, Robustness is patient‐specific. Figures 6 and 7 also show the DVH bands for proton beamlines of two specific patients (patient 2 and patient 10), where robustness for LPB and LPMB is better than that for CPB for patient 2 and CPB robustness is better than that of LPB and LPMB for patient 10.

## 4 DISCUSSION

All dosimetric endpoints improve from VMAT photon and cyclotron‐generated proton plans using the LIGHT nominal system and LIGHT mini beams. In this study, we not only investigated the difference in normal tissue sparing between conventional IMPT plans and photon plans, but we also investigated the plan quality and normal tissue sparing among the different IMPT plans, including three different spot sizes (cyclotron, LIGHT, and LIGHT mini beams). This novelty differentiates our study and makes it unique from other studies that have been performed in the past.

One of the most remarkable results demonstrated by linear accelerator‐generated proton plans in the present study was the significant reduction in normal tissue and OAR doses compared with cyclotron‐generated proton plans and VMAT. Previous studies on conventional (e.g., cyclotron) IMPT for HN cancer vs. photon IMRT have shown improved OAR doses over photon treatments.^10^, ^31^, ^32^, ^33^, ^34^, ^35^, ^36^, ^37^ In a study by Nguyen et al.,^10^ the dosimetric advantages of IMPT compared with VMAT for unilateral HN cancers were compared. This study showed that IMPT held an advantage over IMRT in the defined volumetric doses to the brain stem, spinal cord, optic structures, cochlea, larynx, contralateral parotid, and oral cavity, with a few exceptions. In addition to OAR doses, the authors of the same study also observed an overall advantage in the mean dose to the body and max doses in PTVs. In the present study, we observed a similar result, with CPB plans showing improved OAR doses from VMAT by up to 40%, with the mean dose of the larynx and oral cavity showing the most improvement between VMAT and CPB plans. However, one exception to OAR dose improvement was the mandible, which increased in D_1cc_ by 0.72% from VMAT to CPB plans but also stayed within the recommended dosimetric constraints, so the dose increase seen for CPB plans was not clinically significant. The different anatomic relationship among the OARs and tumors for different patients also contributes to variation in OAR doses for different plans, so some OARs could have received more dose than others for different patients, leading to a significant increase in dose for some OARs, but not others in different cases. LPB and LPMB plans showed an even more remarkable result than VMAT plans, with a 48% and 50% improvement in the mean body dose for LPB and LPMB plans, respectively. The mean dose of the larynx showed the most improvement for LPB and LPMB plans compared to VMAT and CPB plans. All OARs showing major dosimetric improvements between VMAT and proton plans showed significant differences, except for the mandible, between VMAT and all proton plan types. This is most likely because the mandible is often overlapped or adjacent to the target volume and cannot be fully avoided by either photon or proton beams. We did not measure minimum and maximum doses in our study. However, HI showed a significant improvement from VMAT to all proton IMPT plans, demonstrating an improvement in the maximum dose and more homogenous IMPT plans than VMAT plans. In a study by Lewis et al., 10 patients with nasopharyngeal carcinoma were identified for whom IMPT was planned, with nine of the 10 patients comparing photon‐based IMRT. A dosimetric comparison of mean radiation doses to 29 adjacent OARs was also performed. The authors of this study observed the dosimetric advantages conferred by IMPT over IMRT and reported 13 OARs receiving lower mean doses with proton‐based plans. Locoregional control was 100%, and 2‐year overall survival was 88.9% for proton‐based plans. Bilateral HN recurrences have also been reported to be more beneficial using spot‐scanning proton therapy, including therapies like spot‐scanning proton arc therapy (SPArc). Proton linacs are also ideal for SPArc, an advanced IMPT technique that dynamically delivers the proton beam with a rotational gantry,^38^ due to the fast energy changes they are capable of. In a study by Liu et al., SPArc showed a significant sparing of OARs with similar target coverage compared to 3‐field IMPT. The SPArc plans showed equivalent or better robust target coverage while showing significant dosimetric improvements in most OARs. These results are comparable to those presented in the present study using linear accelerator protons. However, one challenge presented for SPArc could be its capability to deliver numerous spots with significantly lower MU weighting than traditional IMPT. Similar studies were performed by Kandula et al.,^35^ who found that spot‐scanning proton therapy could significantly reduce the integral dose to the HN critical structures, and Quan et al.,^36^ who recommended MFO as the preferred planning method for treating bilateral HN cancer due to its fair target coverage and superior sparing of OARs in worst case scenarios. Due to this phenomenon, the MFO planning method was also utilized in the present study.

Spot size in IMPT planning impacts plan quality considerably, which served as one of the most significant differences between CPBs and linear accelerator‐generated proton plans. Plan robustness, conformity, and OAR sparing for proton plans are primarily affected by spot size.^39^ Many past studies comparable to this particular study have observed the effects of large versus small spot‐size machines.^39^, ^40^, ^41^, ^42^, ^43^ However, most of these studies were performed on lung tumors, not HN tumors. Our study demonstrates that as spot size decreases, dose to normal structures and the mean body dose also decrease. Our study is the first to investigate the impact of spot size and using different machine models for HN cancer with bilateral neck irradiation. To show the effects of smaller spot sizes, Liu et al. demonstrated that robust optimization with small‐spot machines significantly improves heart, lung, and esophagus sparing with similar target coverage, plan robustness, and interplay effects compared to plans generated by traditional large‐spot machines. IMPT, using smaller spot sizes, generally produces less robust plans and utilizes more spots to cover the same tumors.^39^ In our study, LPMB plans generally were more challenging in terms of achieving the same robust criteria as LPB and CPB plans, but the final results are comparable to CPB and LPB plans. Given similar tumor dose coverage and prescription doses, the smaller spot machines achieved significantly lower OAR and mean body doses, as also shown by the results of this study, and serve as a substantially better alternative to CPB or VMAT. Plans using minibeams will generally be more robust, but OARs are still within the clinically acceptable ranges. These results could be due to the reduction in penumbra of smaller spot sizes compared to larger spot sizes due to changing spot dimensions.^44^ The small spot size, sharp penumbra, and plan optimization can impact on plan quality and robustness substantially. The proton plans with smaller spots are more sensitive to “interplay effects”, with the dose distribution being significantly affected by small shifts in beam positioning. On the other hand, for the same tumor volumes, smaller spot size requires more spots to treat the tumor volumes and thus enhance the degree of freedom in plan optimization such that it may increase plan robustness. We found in this particular study that robustness is generally better for CPB, however, in Figures 5 and 6, we observe some cases that display better robustness for LPB and LPMB plans. In a study by Widesott et al.,^45^ who assessed plan quality of dose distributions in actual clinical cases for different dimensions of scanned proton pencil beams, authors varied the σ of the initial Gaussian size of the spot from σ*_x_* = σ*_y_* = 3 mm to σ*_x_* = σ*_y_* = 8 mm to evaluate the impact of proton beam size on the quality of IMPT plans. It was determined that acceptable σ*_s_* are ≤ 4 mm for HN tumors. Due to HN patient plans, which deal with complex target volumes and a large number of organs at risk, the authors of this study concluded a small beam is required to be competitive with state‐of‐the‐art photon therapy.^45^, ^46^ In the present study, we confirm these results, showing that a smaller σ results in similar target coverage and lower OAR doses than using a machine model with larger σ_s_. For IMPT, spot dimensions change from one energy layer to the next.^44^ Since HN IMPT plans in the present study used energies with higher weighting towards the middle range of energies, spot sizes for these particular plans were on the lower end of the spot size range, which was ≤4 mm for LPB and LPMB plans. Besides spot size, other factors that could influence plan quality could be the energy ranges on the proton linac machines. As mentioned before, unlike the spot enlargement observed with decreasing energy for cyclotrons, the proton linac spot size is invariant (constant) between energies of 150–230 MeV, rendering proton linac treatment plans more conformal than cyclotron‐based plans, as we can see from the dose distributions presented in Figure 3. Spot sizes for the proton linac become smaller at higher energies, allowing the use of higher energies to treat with at smaller spot sizes.

Regarding IMPT planning, coplanar fields were used because they may encompass a more efficient planning technique for HN cancer with bilateral neck irradiation than non‐coplanar fields. Yi et al. demonstrated no significant differences in plan quality regarding CTV coverage (D_95_) or OAR doses between non‐coplanar and coplanar fields. No substantial changes in the plan robustness were found between non‐coplanar and coplanar fields.^47^ Treatment delivery of non‐coplanar fields is also more complex due to the extra set‐up verification and extra attention to patient safety, which prolongs treatment times. The authors of this particular study by Yi et al. also recommend against using lateral gantry angles (90 or 270 degrees) to avoid a beam path passing through the shoulder and instead recommend using gantry angles of ± 15° from lateral angles while using a coplanar field set‐up. Field angles used in our study were within this range. MFO planning technique was used in IMPT planning due to its ability to optimize multiple fields simultaneously to treat tumors that are hard to reach or surrounded by more sensitive structures. It also allows for superior dose distributions compared with SFO. In a study conducted by Cubillos‐Mesias et al., who studied the impact of robust treatment planning on SFO and MFO plans for proton beam therapy of unilateral HN target volumes, recommended that the MFO technique to ensure plan robustness and reduced median doses to the ipsilateral parotid gland.^48^ Also, in our particular study, very precise settings for spot spacing, energy layer spacing, and lateral margins were used and set to a value of “automatic with scale” for all these parameters. This was done to optimize plan quality and, in the case of lateral margins, to minimize penumbra to a more reasonable level and spare OARs to a greater extent.

This study has a few limitations despite proving the superiority of proton linac plan quality versus cyclotron‐generated proton therapy systems and VMAT. First, the study is based on a proton‐beam line that is not used clinically. Dosimetric accuracy in phantom measurements will be needed to confirm the advantage of mini‐beam protons. Future studies could be expanded to planning for HN cancer with unilateral neck irradiation and different types of HN cancer that are also widely planned in clinical settings, including base of skull plans to determine the effects of using proton linac systems on those particular sites. Sample size of this study was fairly small and more cases could improve the statistics of the study. The study was also performed at a single institution. Expanding this study to multiple institutions would leverage a larger patient population, and results would more closely approximate the “real world” than a set of patients from a single institution. This study investigated how plan quality and normal tissue sparing for HN plans changed using four different techniques: VMAT photon, CPB, LPB, and LPMB. No beam modifiers, such as apertures, were used, which could further improve plan quality and be the topic of a future study. Since robustness was studied in this manuscript, repainting techniques could also be indicated for HN cancer to improve treatment dose delivery, as it is for lung tumors.^49^ Repainting allows the energy layers of the proton beam to be delivered more than once to achieve statistical averaging of motion effects.^49^, ^50^ This could also be the topic of a future study for proton therapy for HN cancers.

Through this particular study, we see how different technology and variation in spot sizes affects plan quality for HN tumors. Proton linacs are capable of producing smaller spot size ranges with a near‐constant penumbra for the clinically useful energy ranges of 150–230 MeV. We anticipate that proton linacs will reduce machine footprint and total cost while improving beam control with a fast energy switch.

## 5 CONCLUSION

With decreased spot size, the LPB and LPMB are excellent alternatives to VMAT and CPB therapy for HN radiotherapy with bilateral neck irradiation. With the benefit of producing treatment plans with lower OAR and normal tissue doses with similar target coverage as VMAT and CPB, they can significantly decrease the dose to normal tissue for bilateral HN cancer radiotherapy treatments.

## AUTHOR CONTRIBUTIONS

Tara Gray was the first author and responsible for conception of the project ideas, overseeing the project, all parts and data in the manuscript as well as writing the manuscript. Chieh‐Wen Liu and Saeed Ahmed were responsible for some treatment planning and obtaining data. Jeremy Donaghue was responsible for providing scripts to obtain data from the treatment planning system. Kevin Stephans, Gregory Videtic, Shlomo Koyfman, Shauna R. Campbell, and Neil Woody were all responsible for contributing knowledge and ideas as well as editing the manuscript. Jonathan B. Farr and Ping Xia were responsible for project funding, conception of the project idea, contributing ideas to and guiding the progress of the project.

## CONFLICT OF INTEREST STATEMENT

Ping Xia and Jonathan Farr both disclose research funding between Cleveland Clinic and Advanced Oncotherapy through the research grant for this work—IRB number 19–1180, DATA1119: Using proton mini‐beams to improve SBRT/SRS plan conformity. Jonathan Farr discloses his position as Journal of Medical Physics (academic journal of the AAPM) Board Member at large—advisory to the editor, his stock in Advanced Oncotherapy plc, and his position as Advanced Oncotherapy plc Chief Clinical Officer A.D.A.M SA Director General.
